# Effects of Non-Indigenous Oysters on Microbial Diversity and Ecosystem Functioning

**DOI:** 10.1371/journal.pone.0048410

**Published:** 2012-10-29

**Authors:** Dannielle S. Green, Bas Boots, Tasman P. Crowe

**Affiliations:** 1 Marine Biodiversity, Ecology and Evolution Group, School of Biology and Environmental Science, University College Dublin, Dublin, Ireland; 2 School of Biosystems Engineering, University College Dublin, Dublin, Ireland; MESC, University of South Alabama, United States of America

## Abstract

Invasive ecosystem engineers can physically and chemically alter the receiving environment, thereby affecting biodiversity and ecosystem functioning. The Pacific oyster, *Crassostrea gigas*, invasive throughout much of the world, can establish dense populations monopolising shorelines and possibly altering ecosystem processes including decomposition and nutrient cycling. The effects of increasing cover of invasive *C. gigas* on ecosystem processes and associated microbial assemblages in mud-flats were tested experimentally in the field. Pore-water nutrients (NH_4_^+^ and total oxidised nitrogen), sediment chlorophyll content, microbial activity, total carbon and nitrogen, and community respiration (CO_2_ and CH_4_) were measured to assess changes in ecosystem functioning. Assemblages of bacteria and functionally important microbes, including methanogens, methylotrophs and ammonia-oxidisers were assessed in the oxic and anoxic layers of sediment using terminal restriction length polymorphism of the bacterial 16S rRNA, mxaF, amoA and archaeal mcrA genes respectively. At higher covers (40 and 80%) of oysters there was significantly greater microbial activity, increased chlorophyll content, CO_2_ (13 fold greater) and CH_4_ (6 fold greater) emission from the sediment compared to mud-flats without *C. gigas*. At 10% cover, *C. gigas* increased the concentration of total oxidised nitrogen and altered the assemblage structure of ammonia-oxidisers and methanogens. Concentrations of pore-water NH_4_^+^ were increased by *C. gigas* regardless of cover. Invasive species can alter ecosystem functioning not only directly, but also indirectly, by affecting microbial communities vital for the maintenance of ecosystem processes, but the nature and magnitude of these effects can be non-linear, depending on invader abundance.

## Introduction

Biological invasions by non-indigenous species are among the most serious threats to biodiversity, ecosystem functioning and the provision of ecosystem services in terrestrial, freshwater and marine environments [1]. In fact, repairing the damage caused by invasive species is estimated to cost nearly 5% of the world’s economy per year [2]. Many of these costs are due to a deterioration of ecosystem services [1], but the costs of prevention, management and mitigation can also be substantial [3]. Such expenditure must be justified and strategically targeted on the basis of robust empirical evidence.

Invasive species can form dense populations, covering large proportions of habitats after successful establishment [4]. The nature and magnitude of their effects on receiving ecosystems may vary spatially and over the course of establishment as a result of variation in abundance [5]. Despite this, knowledge of how their impacts on receiving ecosystems vary with invader abundance is lacking [6], particularly concerning how ecosystem functioning is affected [7]. Studies on the impacts of invasive species typically compare presence or absence of invaders, but in order to improve predictions, it is advantageous to relate invader effects directly to their abundance [6].

There is growing awareness that impacts of invasive species on microbial communities and the processes that they drive are extremely important for the functioning of ecosystems [8]. Invasive species have the potential to modify the diversity or composition, activity or biomass of recipient microbial communities, leading to changes in ecosystem processes and functioning [9]. This may be manifested, for example, as changes in the rates of decomposition and nutrient cycling [10]. Most research has focused on either processes (e.g. biogeochemical flux rates) or, less often, on microbial community characterisation. To gain a true mechanistic understanding of how ecosystem functioning is affected by invasive species, however, we need research that couples ecosystem processes with the characterisation of microbial communities. In addition, studies that measure multiple, rather than single ecosystem processes, are required to assess more fully how ecosystem functioning is affected by invasive species [11].

The effects of invasive species on microbial communities and processes has been extensively researched in terrestrial systems [8], [12], but there is still a dearth of information concerning these effects in marine ecosystems. Coastal ecosystems supply approximately 77% of global ecosystem services [13], such as animal nutrition, organic matter (OM) decomposition, nutrient regeneration and stabilisation of pollutants [14]. Of particular importance in maintaining these services is the role that microbes play, especially in the cycling of nitrogen [15] and carbon [16]. Many microbially mediated processes depend on the redox condition within the sediment [17], with reducing processes predominantly occurring in anoxic sediments. For example, the production of CH_4_ by methanogenic microbes mainly occurs in the anoxic layers of sediments.

Microbial assemblages can be altered through an increased supply of organic matter, or the provision of organic substrates with a different chemical composition from that of pre-invasion conditions. An increase in the cover of bivalves may result in increased deposition of organic matter from faeces and pseudo-faeces. This can affect the microbial decomposer communities and can alter redox conditions in both the oxic and anoxic layers of sediment [18]. These changes may cause an increase in decomposition, leading to increased respiration of CO_2_ and CH_4_ from the sediment.

The Pacific oyster, *Crassostrea gigas*, an invasive bivalve, has spread throughout much of the world and can form very dense reefs covering extensive areas of shoreline [19]. While their effects on the biodiversity of macro-organisms have been extensively documented (e.g. [20]), those on the diversity of microbes and on ecosystem functioning remain largely unexplored.

The aims of this study were to determine the effects of increasing cover of invasive *C. gigas* on ecosystem functioning (carbon and nitrogen cycling), the diversity and community composition of functionally important microbes (methane producers, ammonia-oxidisers) and the biomass of primary producers. It was hypothesised that, due to increased deposition of organic matter from faeces and pseudofaeces resulting from increasing cover of *C. gigas*, (i) carbon and nitrogen flux from the sediment will increase, (ii) primary productivity and microbial activity in the sediment will increase, and (ii) microbial diversity will be affected by increasing cover of *C. gigas*.

## Methods

### Ethics Statement

Informal contact was maintained with the relevant authority, the National Parks and Wildlife Service, who were fully informed of what we were doing and required no formal application for permission to complete the work.

### 2.1 Study Site and Experimental Design

An experiment was set up on an extensive, lower intertidal mud-flat at Ballylin Point, Lough Swilly in County Donegal, Ireland (55° 2′ 36.12′′, −7° 33′ 36.09′′). The experimental mud-flats were not dominated by any other biogenic habitat forming organism or hard substratum but were adjacent to mussel-beds of *Mytilus edulis*. The experiment had one fixed factor, “Cover” with four levels: zero (0%), low (10%), medium (40%) and high (80%) cover of *C. gigas*, which equated to approximately 0, 8, 80 and 150 individual *C. gigas* m^−2^ respectively, equivalent to 0, 26.1±2.1, 390.9±31.9 and 781.8±63.9 g ash free dry weight m^−2^ (mean ± S.E.). There were seven replicate plots, each measuring 50×50 cm, for each cover treatment. Oysters were inserted upright into the sediment to simulate how they were found elsewhere on the shore. All oysters used in this experiment were taken from the surrounding mussel-beds and were rinsed with seawater prior to use to remove attached sediment and flora and fauna. After three months (June 2011) all plots were destructively sampled.

### 2.2 Measuring Ecosystem Processes

#### 2.2.1 Total organic carbon and total nitrogen in the oxic and anoxic sediment

Total organic carbon (TOC) and total nitrogen (TN) were measured using 50 mg of homogenised and pulverised oven dried (80°C for 24 h) sediment after being fumigated for 24 h with HCl vapour to remove carbonates. TOC and TN contents were determined on a Vario EL Cube (Elementar, Germany) and expressed as weight percentages.

#### 2.2.2 Ammonium and total oxidised nitrogen in sediment pore-water

Pore-water was sampled from the sediment-water interface (0 cm), 1 and 4 cm depth using modified Rhizon™ (Rhizosphere, Wageningen, the Netherlands) in-situ profilers [22]. The profilers consisted of Perspex sheets allowing the attachment of Rhizon™ moisture samplers, consisting of 10 cm long filters made of a hydrophilic porous polymer with 0.1 µm pore size. Profilers were carefully inserted into the sediment and left for 24 h prior to sampling in order for the sediment to reach equilibrium. Over-lying surface water (S.W.) (∼2–4 mm above the sediment-water interface) was also collected from each plot. Water samples were transported and stored in separate vacuum tubes. Ammonium (NH_4_^+^) and total oxidised nitrogen (TOxN) were determined using a Lachat Quikchem 8000 (Hach Company, Colorado USA) flow injection auto-analyser. Pore-water nutrient concentrations were corrected for porosity and standardised to dry bulk density. Diffusive fluxes for NH_4_^+^ were calculated from concentration gradients in the pore-water profiles assuming Fick’s first law of diffusion.

#### 2.2.3 Community respiration (CO_2_ and CH_4_)

CO_2_ and CH_4_ were measured using a closed chamber technique based on [21]. This involved placing 6 l airtight, tinfoil covered chambers fitted with rubber septums onto each plot. Gas samples were taken at 0, 45 and 90 min intervals using separate 60 ml syringes fitted with an airtight, three-way stopcock. Air within the chambers was homogenised by gently pumping the syringe three times immediately before samples were taken. Concentrations of CO_2_ and CH_4_ were measured using gas chromatography (Shimadzu GC-2024) with an automated injection system. Certified gas calibration standards (Argo International) were used to create calibration curves ranging from 0 to 5 ppm. Similar standards were also used for quality control after each eight random samples to ensure the quality of analysis was high. Air temperature was measured inside the chambers and atmospheric pressure was estimated after consulting the Met Éireann website (www.meteireann.ie). Flux rates were calculated by assuming the ideal gas law coupled with linear regression, correcting for differences in chamber temperature and average air pressure during the sampling period. An exponential equation, however, was used if R^2^ was greater than 0.985 but less than 1 [21].

Three procedural controls were included to account for (i) the volume taken up by *C. gigas* within the gas chambers, (ii) the respiration of the oysters or (iii) the respiration of macro-organisms attached to their shells. To account for space occupied by *C. gigas*, *C. gigas* valves were glued air-tight, displacing similar volumes of air as live *C. gigas* in the high cover treatment. Additionally, cleaned (to remove flora and fauna) or uncleaned, live *C. gigas* were used to determine direct gas emission from *C. gigas* and associated flora and fauna respectively. These controls were set up simultaneously on the shore using an airtight, impermeable barrier to eliminate respiration from the sediment.

#### 2.2.4 Chlorophyll content of the oxic layer of sediment

The oxic surface layer (approximately the top 2 mm) was sampled using sterile spatulas, immediately wrapped in tin foil to omit light and stored at 4°C. Within 24 h of collection, 10 ml of 90% acetone was added to 1 g of field-moist, homogenised sediment and centrifuged at 3000 g for 3 min. Chlorophyll a, b and c concentrations were measured from the supernatant using a spectrophotometer (λ = 430 and 664, 460 and 647, and 630 nm respectively). Concentrations of chlorophyll in the sediment were calculated according to equations by [23].

#### 2.2.5 Dehydrogenase enzyme activity as an estimate of microbial activity

Dehydrogenase enzyme activity can be used as a measure for microbial activity and was done for both the oxic (top 2 mm) and anoxic (4 cm deep) sediment using a modified triphenyltetrazolium chloride (TTC) method as described by [24], using 1 g of field-moist, homogenised sediment within 24 h of collection. The reaction has triphenyl formazan (TPF) as an end-product which can be estimated colorimetrically (λ = 546 nm). Dehydrogenase activity is reported as µg TPF g^−1^ dry sediment 24 h^−1^. Since the reaction involves aerobic oxidation of the organic substrate, additional measurements were done on the anoxic sediment samples after being autoclaved (121°C, 20 min) and were compared to non-autoclaved sediment.

### 2.3 Microbial Diversity

#### 2.3.1 DNA extraction from the oxic and anoxic sediment

DNA was extracted from 0.5 g of field-moist, homogenised sediment using the method described by [25]. Samples were added to sterile 2 ml polyethylene screw-capped centrifuge tubes, containing 0.5 g of 0.1 mm sterile glass beads (Thistle Scientific), 0.5 g of 0.5 mm sterile zirconium beads (Thistle Scientific) and 0.5 ml modified hexadecyltrimethylammonium bromide (CTAB) extraction buffer (equal volumes of 10% CTAB in 0.7 M NaCl with 240 mM potassium phosphate buffer at pH 8.0). After 10 min incubation at 70°C, 0.5 ml of phenol:chloroform:iso-amylalcohol (25∶24:1) was added. Physical lysis of cells was carried out by bead beating with a Hybraid Ribolyser at 5.5 m s^−1^ for 30 s. Supernatant was collected after centrifugation at 14000 g at 4°C for 5 min and cleaned twice with chloroform:isoamylalcohol (24∶1) to remove impurities. DNA yield was improved by ethanol-precipitation. The resulting DNA was further purified with a PCR product purification kit (Roche) according to manufacturer’s protocol. Presence of DNA was confirmed by electrophoreses on a 1.2% agarose (Roche) gel. DNA concentrations were quantified on a UV spectrophotometer (Nanodrop) and standardised to ∼30 ng µl^−1^ for downstream analyses. Each sample was extracted in triplicate.

#### 2.3.2 Polymerase chain reactions and terminal restriction fragment length polymorphism

All forward primers were fluorescently labelled (detailed in Table S1). Bacterial 16S rRNA was amplified using primer pairs F27 - R1469 [26]; bacterial ammonia-oxidiser amoA, excluding archaeal ammonia-oxidisers, using amoa-1F - amoaR [27]; bacterial methylotroph mxaF using mxa-f1003– mxa-r1561 [28]; and archaeal methanogen mcrA using mlf – mlr [29]. PCR conditions were as detailed in Table S2. For all reactions, PCR was carried out in 50 µl volumes, containing final concentrations of 2.5X PCR buffer (Promega), 30 pM forward and reverse primers (Sigma), 0.25 mg ml^−1^ BSA (New England Biolabs Inc.), 0.2 µM of each dNTP (Sigma), 2.5 µl of ultra clean H2O (Fluka) and 0.125 U of Taq polymerase (Promega). 1 µl of template DNA was added to 24 µl of ultra clean H_2_O prior to adding the PCR mix. PCR products were confirmed on a 1% agarose gel and subsequently purified using a high pure PCR product cleanup kit (Roche) as per manufacturer’s instructions. Approximately 50 ng of PCR product was digested using different restriction endonucleases in final volumes of 20 µl, using buffers at 1x concentrations. 16S rRNA and mcrA fragments were digested at 37°C for 4 h with 20 U of MspI restriction endonuclease (New England Biolabs Inc.). Similarly, mxaF fragments were digested at 37°C for 4 h with 20 U of HhaI restriction endonuclease (New England Biolabs Inc.) and amoA fragments were digested at 65°C for 4 h with 20 U of TaqI restriction endonuclease (New England Biolabs Inc.). The digested products were desalted and cleaned by precipitation in ethanol. The differently labelled digests were pooled for each separate sample, and 2 µl of this was mixed with 0.5 µl 600LIZ size standard (Applied Biosystems) and 9.0 µl formamide loading solution (Applied Biosystems). Terminal restriction lengths were analysed by electrophoresis using a 36 cm capillary for 30 min at 8 kV on a 3031 ABI Genetic Sequencer (Applied Biosystems). Fragment analysis was done using Genemapper (Applied Biosystems). Threshold levels for peak detection were set at 20 rfu, and peaks were called using a quartic polynomial model. Microbial assemblage profiles obtained were sorted and aligned using RiboSort in R [30]. Only fragments that contributed more than 1% of the total abundance (rfu) of each sample were considered true fragments.

### 2.4 Statistical Analyses

Differences in gas fluxes of CO_2_ and CH_4_, diffusive flux of NH_4_^+^, concentrations of chlorophyll a, b and c, TOC, TN, C:N ratio, microbial activity and the number of restriction fragments among different covers of *C. gigas* were evaluated using one-factor (“Cover”) ANOVA with four levels (zero, low, medium and high) using Win-GMAV [31]. Procedural controls for gas fluxes were evaluated using a one-factor ANOVA with four levels (volume, oyster and macro-organism controls and high cover plots). Pore-water concentrations of NH_4_^+^ and TOxN were analysed using a two-factor ANOVA with “Cover” and “Depth” of the profile. Homogeneity of univariate variance was tested using Cochran’s C-test. When this was significant, data was square root transformed to decrease the probability of a Type I error [32]. When significant differences were detected by ANOVA (at α = 0.05), Student-Newman-Keuls (SNK) tests were done to compare means and identify patterns of difference.

Differences in microbial assemblage structure between different covers of *C. gigas* were assessed using one-factor PERMANOVA [33] in the oxic and anoxic sediment separately on Bray-Curtis dissimilarities [34] of 4^th^ root transformed data with 9999 permutations of raw data. Assemblages were ordinated using two-dimensional non-metric multidimensional scaling (nMDS), with the stress values representing the level of distortion of the actual rank order of distances among samples [35]. All multivariate analyses were computed using the PRIMER v6 and PERMANOVA+ add-on package (PRIMER-e, Plymouth, UK).

## Results

### 3.1 Carbon and Nitrogen in the Sediment

Although total organic carbon and total nitrogen increased slightly with increasing cover of *C. gigas* in the oxic layer, the concentration of total organic carbon, total nitrogen and C:N ratios did not differ significantly among different covers of *C. gigas* within the oxic or anoxic sediment (Table 1).

**Table 1:** Concentration of total organic carbon (%), nitrogen (%) and C:N ratio of the oxic and anoxic sediment layers with increasing cover of *C. gigas*.

Layer	Cover	C	N	C:N
Oxic	Zero	0.96±0.12	0.15±0.01	6.23±0.33
	Low	0.86±0.04	0.15±0.01	5.78±0.29
	Medium	0.93±0.05	0.17±0.01	5.65±0.35
	High	1.15±0.11	0.18±0.01	6.15±0.22
ANOVA	df1 = 3,	F = 2.14,	F = 2.46,	F = 1.07,
	df2 = 24	*P* = 0.122	*P* = 0.087	*P* = 0.380
Anoxic	Zero	1.10±0.09	0.16±0.01	6.84±0.37
	Low	1.06±0.06	0.14±0.01	7.51±0.34
	Medium	1.04±0.04	0.15±0.01	6.80±0.23
	High	1.04±0.06	0.15±0.01	6.82±0.34
ANOVA	df1 = 3,	F = 1.66,	F = 0.23,	F = 1.32,
	df2 = 24	*P* = 0.202	*P* = 0.875	*P* = 0.291

Different letters indicate significant differences between covers within each layer, df  =  degrees of freedom. Mean ± S.E., n = 7.

**Figure 1:**
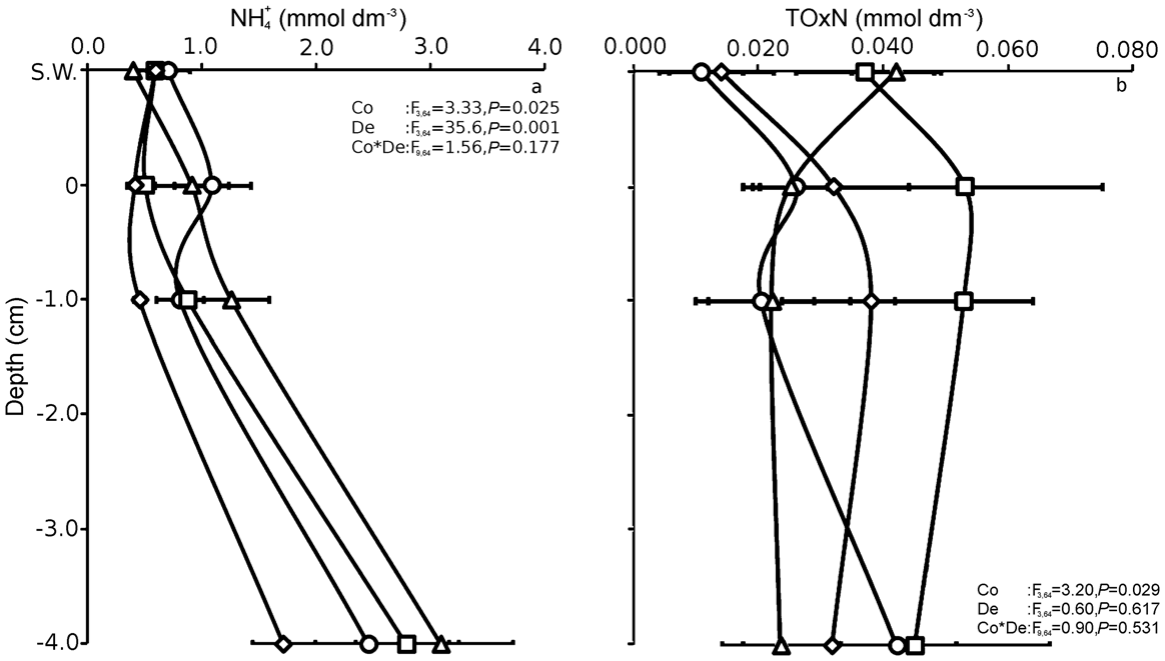
Concentration (mmol g^−1^ dry sediment) of (a) ammonium and (b) total oxidised nitrogen of surface water (S.W.) down to 4 cm depth in the sediment with zero (◊), low (□), medium (Δ) or high (○) cover of *C. gigas.* ANOVA F-ratios and *P*-values are included with the factors Cover (Co) and Depth (De). Mean ± S.E., n = 7.

NH_4_^+^ concentration in the sediment profile increased with depth into the sediment and was significantly greater at low, medium or high cover of *C. gigas* than at zero cover (Fig. 1a). The greatest NH_4_^+^ concentration occurred at medium cover of *C. gigas* in the deeper layers (1–4 cm) ranging from 1.25*±*0.33 to 3.07±0.63 mmol dm^−3^. The diffusive flux of NH_4_^+^ was also greater at medium cover (0.27±0.10 mmol m^−2^ day^−1^) compared to zero (0.07±0.05 mmol m^−2^ day^−1^), low (0.10±0.04 mmol m^−2^ day^−1^) or high (0.07±0.04 mmol m^−2^ day^−1^) cover of *C. gigas*, but was only significantly greater in plots with medium compared to plots with zero cover of *C. gigas* (F_3,16_ = 4.16, *P* = 0.02). The concentration of total oxidised nitrogen (TOxN) at low cover of *C. gigas* was significantly greater than that at zero, medium or high cover regardless of depth (Fig. 1b). Interestingly, the concentration of TOxN at medium cover decreased in the deeper layers, whereas it increased at the zero, low or high covers.

### 3.2 CO_2_ and CH_4_ Respiration from the Sediment and Procedural Controls

**Figure 2:**
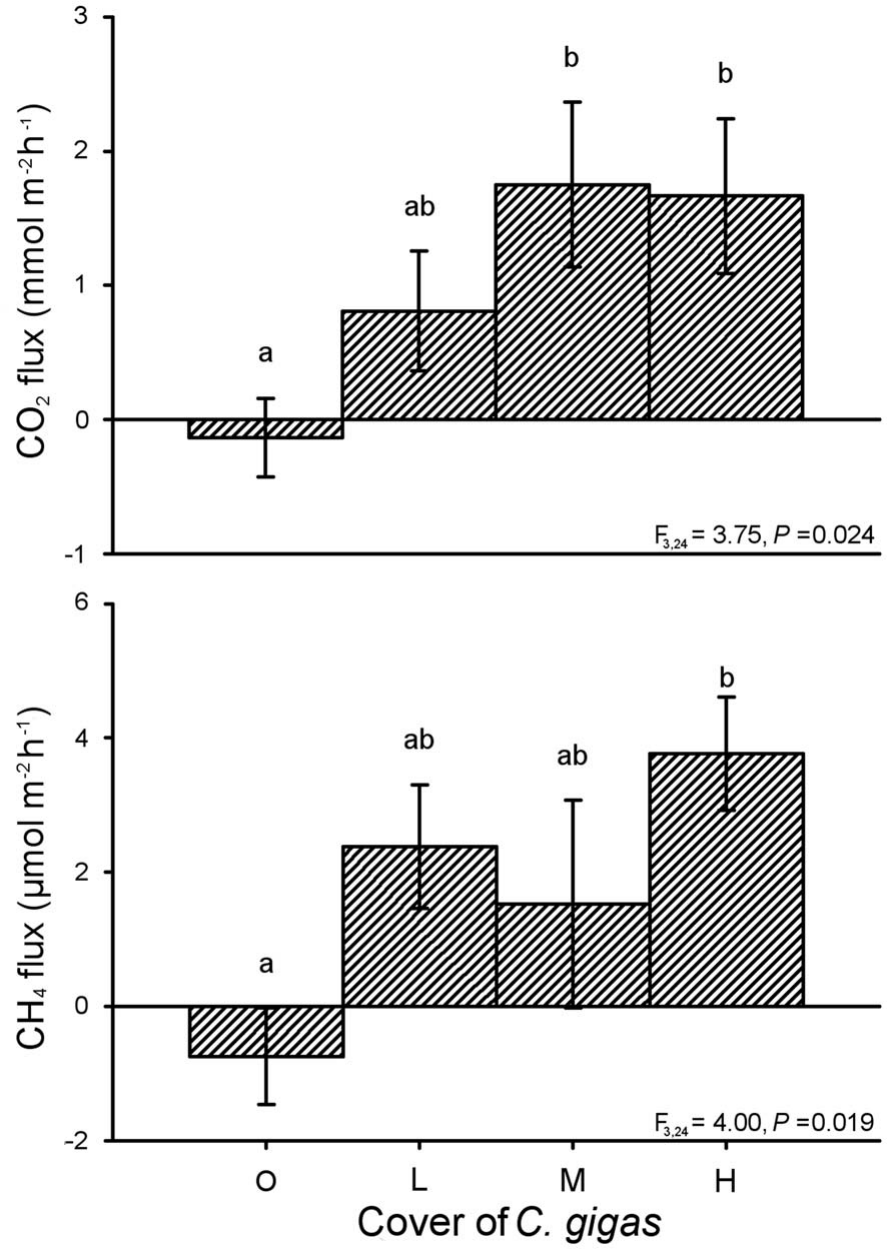
Flux of (a) CO_2_ (mg m^−2^ h^−1^) and (b) CH_4_ (µg m^−2^ h^−1^) from the surface of the sediment with zero, low, medium or high cover of *C. gigas*. Different letters indicate significant differences between covers at *P*<0.05, ANOVA F-ratios and P-values are included. Mean ± S.E., n = 7.

Significantly more CO_2_ was respired from the sediment at higher covers of *C. gigas* (Fig. 2a), with 13 times more CO_2_ emitted from high cover compared to zero cover. The flux of CO_2_ was significantly greater at both medium (77.0*±*22.8 mg m^−2^ h^−1^) and high cover (73.3*±*25.4 mg m^−2^ h^−1^) of *C. gigas* compared to zero cover (−6.0*±*12.8 mg m^−2^ h^−1^). Similarly, more CH_4_ was respired at high cover (60.4*±*13.6 µg m^−2^ h^−1^) of *C. gigas* compared to zero cover (−11.9*±*11.6 µg m^−2^ h^−1^) (Fig. 2b), which equated to a 6 fold increase of CH_4_ respired. In fact, when no *C. gigas* were present, a negative CO_2_ and CH_4_ flux was observed, albeit not significantly different from zero (t = 0.8, *P* = 0.5 and t = 0.3, *P* = 0.8). Fluxes of CO_2_ and CH_4_ from plots with high cover of *C. gigas* were significantly greater than those from volume, oyster or macro-organism controls (F_3,24_ = 6.27, *P* = 0.008 and F_3,24_ = 6.98, *P* = 0.006, for CO_2_ and CH_4_ respectively). This confirms that fluxes originated from the sediment, since neither the volume of *C. gigas*, their respiration nor the respiration of the organisms on their shells contributed significantly to gas emissions (Table S3).

### 3.3 Primary Productivity and Microbial Activity in the Sediment

**Figure 3:**
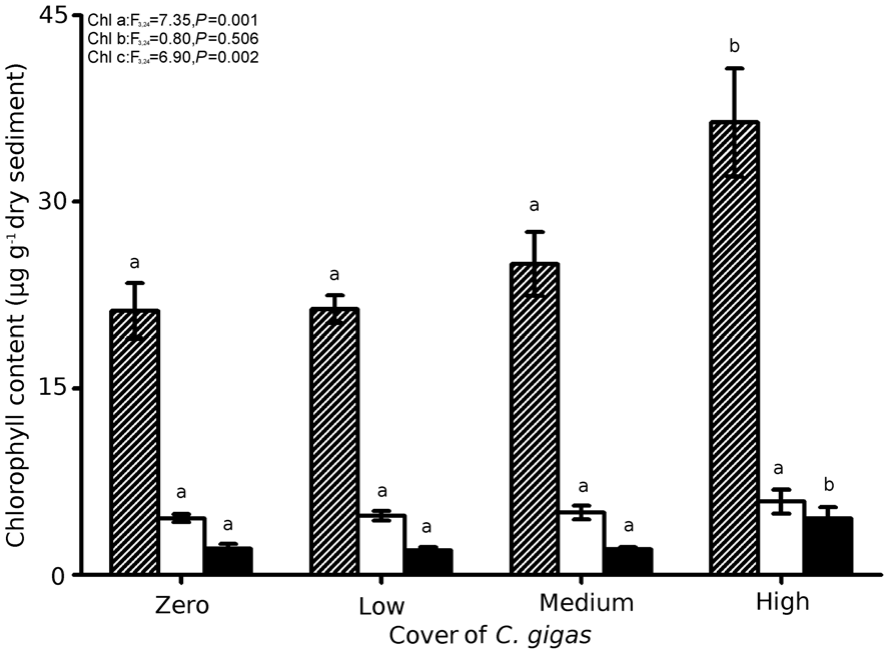
Concentration (µg g^−1^ dry sediment) of chlorophyll a (hashed), chlorophyll b (clear) and chlorophyll c (filled) with zero, low, medium or high cover of *C. gigas.* ANOVA F-ratios and *P*-values are included for chlorophyll a (chl a), chlorophyll b (chl b) and chlorophyll c (chl c) and different letters indicate significant differences between cover at *P*<0.05. Mean ± S.E., n = 7.

The greatest concentration of chlorophyll was found at high cover of *C. gigas* (Fig. 3), characterised by significantly more chlorophyll a and c compared to zero, low or medium covers. The concentration of chlorophyll b was similar regardless of *C. gigas* cover.

Microbial activity, as measured by dehydrogenase activity, was greater at high cover of *C. gigas* compared to sediment with zero, low or medium covers in the oxic sediment (Table 2). Within the anoxic sediment, however, microbial activity was not significantly different among any cover of *C. gigas* (Table 2), corrected for the anoxic conditions of the sediment after autoclaving.

**Table 2:** Microbial activity (µg TPF g^−1^ dry sediment 24 h^−1^) and diversity of total bacteria (S_16S_), ammonia-oxidising bacteria (S_amoA_), methanogenic archaea (S_mcrA_) and methylotrophic bacteria (S_mxaF_) in the oxic and anoxic sediment layers under increasing cover of *C. gigas*.

Layer	Cover	Mic. act.†	S_16S_	S_amoA_	S_mcrA_	S_mxaF_
Oxic	Zero	143±14^a^	29.0±1.9	21.7±1.2^ab^	4.0±0.5	15.4±0.4
	Low	122±4^a^	28.6±1.8	21.3±2.2^ab^	4.0±0.6	15.3±0.5
	Medium	166±18^a^	28.0±1.4	20.6±0.9^a^	4.0±0.7	15.1±0.7
	High	336±76^b^	31.0±2.2	27.4±2.1^b^	3.9±0.6	14.9±0.3
ANOVA	df1 = 3,	F = 6.14,	F = 0.51,	F = 3.45,	F = 0.02,	F = 0.22,
	df2 = 24	***P*** ** = 0.003**	*P* = 0.679	***P*** ** = 0.032**	*P* = 0.989	*P* = 0.880
Anoxic	Zero	589±111	26.0±2.1	18.7±2.5	7.0±0.8	13.7±1.5
	Low	485±37	26.0±3.6	18.0±1.8	8.7±0.6	14.1±0.5
	Medium	515±49	24.4±3.0	16.4±0.6	8.0±1.2	12.6±0.8
	High	661±104	25.0±1.6	17.3±0.9	7.1±1.1	12.0±0.8
ANOVA	df1 = 3,	F = 0.93,	F = 0.08,	F = 0.36,	F = 1.10,	F = 0.67,
	df2 = 24	*P* = 0.443	*P* = 0.970	*P* = 0.782	*P* = 0.368	*P* = 0.579

† Microbial activity.

Different letters indicate significant differences between covers within each layer, df  =  degrees of freedom. Mean ± S.E., n = 7.

### 3.4 Microbial Diversity in the Oxic and Anoxic Sediment

Overall, 89 of bacterial 16S rRNA, 26 of archaeal methanogen mcrA, 55 of bacterial methylotroph mxaF and 131 of bacterial ammonia-oxidiser amoA fragments (as an estimate for diversity) were found. The number of amplified fragments, however, did not significantly differ among different covers of *C. gigas* for total bacteria, methanogen or methylotroph assemblages (Table 2), but ammonia-oxidiser assemblages in the oxic sediment at high cover had greater diversity than that at zero, low or medium covers of *C. gigas* (Table 2).

**Figure 4:**
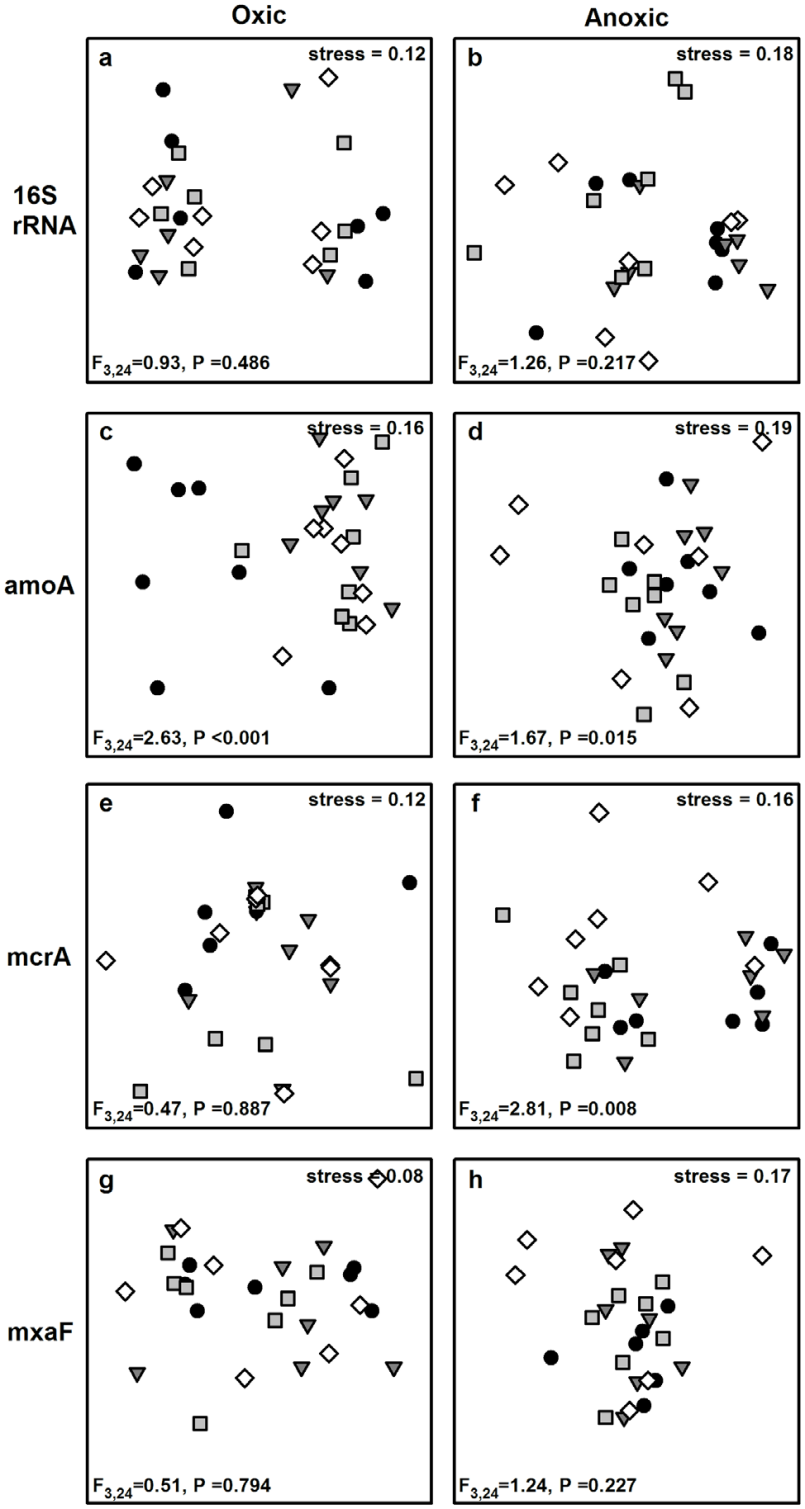
Non-metric multidimensional scaling of assemblages of total bacteria (16S), bacterial ammonia oxidisers (amoA), archaeal methanogens (mcrA) and bacterial methylotrophs (mxaF) in the oxic and anoxic sediment with zero (◊), low (▪), medium (▾) or high (•) cover of *C. gigas*. Pse. udo-F ratios and *P*-values (based on 9999 permutations) obtained from PERMANOVA are included.

Assemblage structure of total bacteria and methylotrophs did not differ among different covers of *C. gigas* in either the oxic or anoxic sediment (Fig. 4a, b, g and h). However, assemblage structure of ammonia-oxidisers in the oxic sediment at high cover of *C. gigas* differed to that at zero, low or medium cover (Fig. 4c). In the anoxic sediment ammonia-oxidiser assemblage structure at low cover of *C. gigas* differed from that at zero, medium or high cover (Fig. 4d). Assemblage structure of methanogens at low cover of *C. gigas* differed from that at zero, medium or high cover (Fig. 4f) in the anoxic sediment, but not in the oxic sediment (Fig. 4e).

## Discussion

### 4.1 Effects of *C. gigas* on Nutrient Cycling and Microbial Assemblages

The link between invasive species, microbial diversity and nutrient cycling in the marine environment has not been explicitly explored until now. Nutrient cycling and microbial assemblage structure and diversity were altered, but the nature and magnitude of effects were dependent on the cover of *C. gigas*. Interestingly, at the highest cover of *C. gigas*, emissions of CO_2_ and CH_4_ were 13 and 6 fold greater compared to mud-flat without *C. gigas*. The procedural controls indicated that the CO_2_ and CH_4_ measurements were not biased by the volume occupied by the oysters in the chamber, nor from their own respiration or the respiration of macro-organisms attached to their shells. This suggests that the increase in CO_2_ and CH_4_ fluxes resulted from the influence of *C. gigas* on processes in the sediment. The abundance of infauna within similar mud-flats has been found to increase with increasing cover of *C. gigas* previously (Green and Crowe, unpublished data). On average, however, the contribution to sediment respiration of meiofauna is negligible, and that of macrofauna is between 10 and 30% [36], so the majority (at least 70%) of the carbon flux measured here can be attributed to microbially mediated processes in the sediment [36].

The greater carbon emission from the plots at the highest cover of *C. gigas* suggests that total carbon catabolism has been altered [37]. Oysters can increase organic matter content locally by increased deposition either directly due to biodeposits [38], but also indirectly, as a result of their shell structure, which can enhance sedimentation rates of particulate organic matter [39]. There was only slight evidence for enrichment of total organic carbon in either the oxic or anoxic sediment, suggesting that rather than being sequestered, the majority of the additional organic carbon was rapidly decomposed by the microbial community, as is evident from increased sediment CO_2_ and CH_4_ emissions.

Causal links between changes to the diversity or structure of microbial assemblages and alterations to ecosystem process rates are possible, and even probable [8], [40], but require further investigation. In the current study, however, there was no effect of *C. gigas* on the diversity or assemblage structure of total bacteria, indicating that a change of microbial diversity did not account for the greater release of CO_2_. Rather, it is likely that greater decomposition rates at the highest cover of *C. gigas*, as suggested by greater levels of microbial activity, accounts for the increased CO_2_ release. Indeed, microbes displaying greater activity and respiration at higher levels of additional labile carbon is a common occurrence [36].

Since gas measurements were done under dark conditions, algal respiration may have contributed to the greater CO_2_ flux [41]. At the highest cover of *C. gigas*, there was an increase in chlorophyll a and c, indicating an increase in the biomass of microphytobenthos [42]. However, this only occurred at the highest cover of *C. gigas*, whereas CO_2_ flux was similar at medium and high cover. There was, however, a greater emission of CH_4_ from the highest cover of *C. gigas* compared to at medium cover. About one third of CH_4_ is produced by the reduction of CO_2_ [43], and an increase in CH_4_ production may explain the CO_2_ flux being similar at medium and high cover. The greater CH_4_ flux may also indicate higher rates of anaerobic decomposition of OM, resulting in more methane production [44] due to a temporary increase in available buried OM from *C. gigas* biodeposits [18]. Alternatively, the increase in CO_2_ and CH_4_ emissions may have been stimulated by the “priming effect” [45], whereby the addition of fresh labile OM (such as from biodeposits) temporarily stimulates microbial decomposition, including that of older, buried, recalcitrant OM. The labile OM necessary to cause a priming effect in aquatic ecosystems is present in microphytobenthos [46] which are commonly present in, or enhanced by, oyster biodeposits.

Methanogen assemblages in the anoxic sediment were significantly different at low cover of *C. gigas*, compared to those at zero, medium or high cover. There is growing evidence suggesting that the assemblage structure of microbial communities influences their functioning [47]. However, no relationship was found between differences in methane emissions and methanogen assemblage structure, which suggests that enhanced activity rather than a change in diversity and assemblage structure accounted for the increased CH_4_ emission. The process of methane-oxidation is dependent on gradients of CH_4_ concentration in the sediment [48], the quality of organic matter [49] and the position of the oxic-anoxic interface [50], all of which can be altered by bivalves [38]. CH_4_ is used by many methane-oxidising bacteria, including some methylotrophs, [51] which typically occur in oxic sediment and regulate the flux of CH_4_ to the atmosphere or water column [52]. The primers used to amplify the mxaF gene [28] do not exclusively represent methane-oxidisers. Nonetheless, this approach still provides information on groups associated with the oxidation of CH_4_ within the sediment [53]. However, neither the diversity nor assemblage structure of methylotrophs was altered by *C. gigas*.

Oyster biodeposits can also enrich the sediment with nitrogen [38] affecting processes such as methanogenesis or methanotrophy and thereby altering carbon cycling. For example, CH_4_ formation by methanogens can be inhibited by competition for OM by sulphate-reducing microbes [54], but the addition of nitrogen can alleviate this and suppress the oxidation of CH_4_ by methanotrophs [55] thereby reducing the loss of CH_4_. In a recent study [56] multiple levels of nitrogen added to marine sediments were found to increase CH_4_ production with increased nitrogen additions, but there were no effects on the community structure, diversity or activity of methanotrophs.

Coastal sediments are critical for global nitrogen cycling and a complex interplay between nitrification, denitrification, and ammonification occurs at the oxic-anoxic interface, driving rapid nitrogen transformations, resulting in the loss of nitrogen to the water column and atmosphere [15], [57]. *C. gigas* altered nitrogen cycling in the current study, while there was only a slight increase of total nitrogen in the sediment with increasing cover of *C. gigas*, the concentration of pore-water NH_4_^+^ increased. This may also indicate an increase in remineralisation of OM [58] likely from increased biodeposition, mirroring findings from the carbon cycle. Diffusive fluxes of NH_4_^+^ changed from negative to positive at low and medium cover of *C. gigas*, compared to plots without *C. gigas*. These fluxes were, however, greatest at medium, rather than at high cover, as might be expected. This discrepancy may be due to the greater levels of microphytobenthos biomass found at high cover of *C. gigas*. Actively growing microphytobenthos can intercept, absorb and assimilate NH_4_^+^ from the surface layers of the sediment, thereby limiting its release to the water column [18], [41]. Bacterial ammonia-oxidising assemblages in oxic sediments at high cover of *C. gigas* were more diverse and had a different structure from those at zero, low or medium cover. The primers used in this study to assess the diversity of ammonia-oxidisers targeted bacterial amoA genes, but sediments may also contain large populations of archaea, which recently have been found to also play significant roles in the oxidation of ammonia [15] and may have responded differently to increasing covers of *C. gigas*. Nevertheless, increased diversity of ammonia-oxidisers has been suggested to increase the stability of nitrification in waste water treatment plants leading to enhanced removal of NH_4_^+^ [59], we cannot determine, however, whether this was the case in this study. Alternatively, others have found no correlation between nitrification rates and the composition of ammonia-oxidiser communities [60]. Although concentrations of total oxidised nitrogen (TOxN) overall were small in the pore-water, values were greatest at low cover of *C. gigas*. This co-occurred with different assemblage structure of ammonia-oxidisers in the anoxic sediment at low cover of *C. gigas* compared to that at medium or high cover. A greater efficiency of nitrification at the low cover, may have resulted in an increase of TOxN concentration, but this could not be determined directly.

### 4.2 Potential Further Implications of *C. gigas* Invasions

The influence of high cover of *C. gigas* increased total carbon fluxes from the sediment at low tide. Recently, Lejart et al. 2012 [61] measured the aerial and underwater respiration of *C. gigas* in laboratory conditions to estimate their direct contribution to the CO_2_ emissions of the Bay of Brest during a whole tidal cycle. They concluded that, at high tide, *C. gigas* (due to its respiration and calcification) contributed significantly to carbon fluxes of coastal ecosystems but that its effects at low tide were negligible [61]. They did not, however, take into account any indirect influences of *C. gigas* on CO_2_ emissions from the sediment as was done in the current study. It is not uncommon for *C. gigas* to dominate large areas of shorelines [19]. At larger scales, such as estuaries, greater emissions of greenhouse gases arising both directly and indirectly due to *C. gigas*, should be considered for inclusion in carbon budget models [62] and warrant further investigation. The impacts of invasive species are, however, often context-dependent [5], [6] and since the current study was only done at a single location and at one time during summer, it is limited in its ability to predict impacts on ecosystem functioning and greenhouse gas emissions at larger spatial or temporal scales. Nonetheless, the current study indicates that even when present at low cover, *C. gigas* can alter ecosystem processes including decomposition and nutrient cycling. The assemblage structure and diversity of microbes involved in decomposition were also altered by *C. gigas*, possibly leading to subsequent changes in decomposition and nutrient cycling within the system [63]. Further research is required, however, to determine the extent to which changes in microbial assemblages affect ecosystem processes. Investigation of microbial responses to invasions can illuminate the mechanisms underpinning functional changes induced by biological invasions. This can be aided by experiments manipulating a range of invader abundances, helping to develop quantitative estimates of future effects and therefore aid in making decisions about appropriate management or mitigation strategies. An interdisciplinary approach was taken in this study involving ecology, biogeochemistry and microbiology. Such an approach is necessary in order to advance knowledge of the important functional roles played by microbes, and to understand how they may be impacted by anthropogenic stressors.

## Supporting Information

Table S1 **Forward and reverse primer pairs used in this study to target bacterial (16S rRNA) and functional genes for ammonia-oxidisers (amoA), methanogens (mcrA) and methylotrophs (mxaF) in the oxic and anoxic sediment.**
(DOCX)

Table S2 **PCR cycles used to amplify bacterial (16S rRNA) and functional genes for ammonia-oxidisers (amoA), methanogens (mcrA) and methylotrophs (mxaF).** All amplifications started with a 2 min denaturing at 95°C and a final extension for 5 min at 72°C.
(DOCX)

Table S3 **CO2 (mmol m-2 h-1) and CH4 (µmol m-2 h-1) from procedural controls (volume, live oysters and macrofaunal) and high cover plots of C. gigas. Mean ±S.E., n = 7.**
(DOC)
