# Supplementary material for: Effects of Non-Indigenous Oysters on Microbial Diversity and Ecosystem Functioning
Source: PLoS One. 2012 Oct 29;7(10):e48410. doi: 10.1371/journal.pone.0048410 (PMC3483273; doi:10.1371/journal.pone.0048410)
Supplement: Table S1 — Forward and reverse primer pairs used in this study to target bacterial (16S rRNA) and functional genes for ammonia-oxidisers (amoA), methanogens (mcrA) and methylotrophs (mxaF) in the oxic and anoxic sediment. (DOCX) [file pone.0048410.s001.docx]

**Supplementary information**

**Table S1.** Forward and reverse primer pairs used in this study to target bacterial (16S rRNA) and functional genes for ammonia-oxidisers (amoA), methanogens (mcrA) and methylotrophs (mxaF) in the oxic and anoxic sediment.

| Target | Name | Primer sequence '5-'3 | Label* |
| --- | --- | --- | --- |
| 16S rRNA | F27 | AGAGTTTGATC(C/A)TGGCTCAG | NED |
|  | R1469 | ACGG(C/T)TACCTTGTTACGACT |  |
| amoA | amoa1-F | GGGGTTTCTACTGGTGGT | 6FAM |
|  | amoaR | CCCCTCKGSAAAGCCTTCTTC |  |
| mxaF | mxa-f1003 | GCGGCACCAACTGGGGCTGGT | PET |
|  | mxa-r1561 | GGGCAGCATGAAGGGCTCCC |  |
| mcrA | mlf | GGTGGTGTMGGATTCACACARTAYGCWACAGC | VIC |
|  | mlr | TTCATTGCRTAGTTWGGRTAGTT |  |

* Fluorescent labels were attached to the '5 end of the forward primer
