# Supplementary material for: Effects of Non-Indigenous Oysters on Microbial Diversity and Ecosystem Functioning
Source: PLoS One. 2012 Oct 29;7(10):e48410. doi: 10.1371/journal.pone.0048410 (PMC3483273; doi:10.1371/journal.pone.0048410)
Supplement: Table S2 — PCR cycles used to amplify bacterial (16S rRNA) and functional genes for ammonia-oxidisers (amoA), methanogens (mcrA) and methylotrophs (mxaF). All amplifications started with a 2 min denaturing at 95°C and a final extension for 5 min at 72°C. (DOCX) [file pone.0048410.s002.docx]

**Table S2.** PCR cycles used to amplify bacterial (16S rRNA) and functional genes for ammonia-oxidisers (amoA), methanogens (mcrA) and methylotrophs (mxaF). All amplifications started with a 2 min denaturing at 95°C and a final extension for 5 min at 72°C.

| Target | Denaturing | Annealing | Extension | Cycles |
| --- | --- | --- | --- | --- |
| 16S rRNA | 95°C - 30 sec | 55°C - 90 sec | 72°C - 90 sec | 26x |
| amoA | 95°C - 30 sec | 45°C - 45 sec | 72°C - 30 sec | 35x |
| mxaF | 95°C - 60 sec | 55°C - 60 sec | 72°C - 60 sec | 30x |
| mcrA^†^ | 95°C - 30 sec | 55°C - 30 sec | 72°C - 90 sec | 30x |

^†^mcrA primers (mlf-mlr) were degenerate, therefore following the 2 min denaturing step, DNA was amplified with an initial 5 cycles of 95°C for 30 sec followed by 45°C for 30 sec with an increasing 0.1°C/sec to 72°C for 90 sec to allow for mismatches prior continuing the program in the table.
