# Supplementary material for: Effects of Non-Indigenous Oysters on Microbial Diversity and Ecosystem Functioning
Source: PLoS One. 2012 Oct 29;7(10):e48410. doi: 10.1371/journal.pone.0048410 (PMC3483273; doi:10.1371/journal.pone.0048410)
Supplement: Table S3 — CO2 (mmol m-2 h-1) and CH4 (µmol m-2 h-1) from procedural controls (volume, live oysters and macrofaunal) and high cover plots of C. gigas. Mean ±S.E., n = 7. (DOC) [file pone.0048410.s003.doc]

**Table S3.** CO_2_ (mmol m^-2^ h^-1^) and CH_4_ (µmol m^-2^ h^-1^) from procedural controls (volume, live oysters and macrofaunal) and high cover plots of *C. gigas*. Mean ±S.E., n = 7.

|  | Volume | Oyster | Macrofaunal | High |
| --- | --- | --- | --- | --- |
| CO_2_ flux | -0.52±0.60 | 0.21±0.67 | 0.74±0.27 | 2.54±0.52 |
| CH_4_ flux | -0.66±0.69 | -0.49±0.59 | 0.01±1.16 | 4.34±1.03 |
